# Enantiospecific Desorption Triggered by Circularly Polarized Light

**DOI:** 10.1002/anie.201906630

**Published:** 2019-09-03

**Authors:** Farinaz Mortaheb, Katrin Oberhofer, Johann Riemensberger, Florian Ristow, Reinhard Kienberger, Ulrich Heiz, Hristo Iglev, Aras Kartouzian

**Affiliations:** ^1^ Catalysis Research Center and Chemistry Department Chair of Physical Chemistry Technische Universität München Lichtenbergstr. 4 85748 Garching Germany; ^2^ Physik-Department E11 Technische Universität München James-Franck-Str. 1 85748 Garching Germany

**Keywords:** anisotropy factor, circular dichroism, enantioseparation, laser desorption, racemic films

## Abstract

The interest in enantioseparation and enantiopurification of chiral molecules has been drastically increasing over the past decades, since these are important steps in various disciplines such as pharmaceutical industry, asymmetric catalysis, and chiral sensing. By exposing racemic samples of BINOL (1,1′‐bi‐2‐naphthol) coated onto achiral glass substrates to circularly polarized light, we unambiguously demonstrate that by controlling the handedness of circularly polarized light, preferential desorption of enantiomers can be achieved. There are currently no mechanisms known that would describe this phenomenon. Our observation together with a simplified phenomenological model suggests that the process of laser desorption needs to be further developed and the contribution of quantum mechanical processes should be revisited to account for these data. Asymmetric laser desorption provides us with a contamination‐free technique for the enantioenrichment of chiral compounds.

## Graphical abstract

**Light fantastic**: By applying right (RCP) or left (LCP) circularly polarized light to racemic samples of 1,1′‐bi‐2‐naphthol (BINOL) coated onto achiral glass substrates, preferential desorption of enantiomers can be achieved, thereby providing contamination‐free enantioenrichment. There are no known mechanisms for this phenomenon, and a simplified phenomenological model suggests that the contribution of quantum mechanical processes should be revisited.

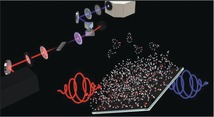

The separation of enantiomers is of great interest since more than 50 % of pharmaceutically active ingredients are chiral, and nine of the top 10 drugs with respect to worldwide sales value have chiral active ingredients. Although they have the same atomic connectivity, enantiomers of most chiral ingredients exhibit markedly different biological activities. Therefore, it is important to promote the enantiomeric enrichment of racemic drugs in order to reduce the amount or eliminate the inactive or even harmful enantiomer in the mixture.^1^ Accordingly, the industrial need for affordable enantioenrichment methods^2^ and the academic demand for high‐resolution studies of chiral molecules in the gas phase^3^ and at surfaces,^4^ have led to a rapid growth in interest in laser desorption (LD).^5^ Herein, we show that by controlling the handedness of circularly polarized light, and hence introducing a gradient between the excitation of the enantiomers, preferential desorption of enantiomers from achiral surfaces can be achieved. This observation suggests that LD can be applied as an additive‐free enantioenrichment method. In our report, racemic BINOL coated onto achiral optical borosilicate glass substrates (BK7) were chosen as a model system for the enantioselective desorption by circularly polarized light.

Absorption and circular dichroism (CD) spectra of racemic, (*R*)‐ and (*S*)‐BINOL in solution are presented in Figure 1 a and b, respectively. The strong UV absorption of BINOL extends up to 350 nm, and the enantiomers show optical activity throughout the depicted range. We prepared the samples by evaporating racemic BINOL onto clean BK7 substrates in a high‐vacuum chamber to form films with controlled thickness (for experimental details, see the Supporting Information). We irradiated the samples with circularly polarized sub‐50 fs laser pulses with a central wavelength of 600 nm and 650 nm, that is, above the onset of the resonant two‐photon absorption (TPA) of BINOL as extracted from second harmonic generation spectrum of BINOL films (see the Supporting Information), in order to desorb intact and neutral molecules,^6^ as confirmed by time‐of‐flight mass spectrum of desorbed BINOL (see the Supporting Information). The inset of Figure 2 shows a microscopy image of the sample region irradiated at 650 nm for 2 hours. The increased transparency indicates the partially desorbed area of the BINOL film.

**Figure 1:**
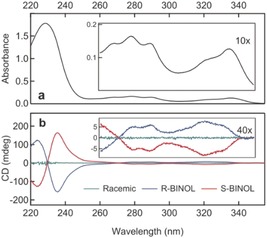
a) Absorption spectrum of BINOL. b) CD spectra of (*R*)‐BINOL (blue), (*S*)‐BINOL (red), and racemic BINOL (green) in ethanol.

**Figure 2:**
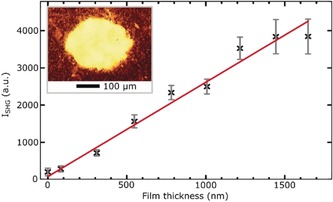
SHG signal measured in BINOL samples with various film thicknesses between 100 and 1700 nm. Inset: Microscopy image of the sample region after LD with 650 nm for 2 hours.

Given the small chiroptical response that is expected from a micron‐scale molecular film, we used second harmonic generation circular dichroism (SHG‐CD) with a chiroptical sensitivity of at least three orders of magnitude higher than its linear counterpart.^7^ Irradiation of the samples as well as the SHG‐CD measurements were performed under atmospheric pressure. Data measured for films with various thicknesses show that the SHG intensity varies almost linearly with film thickness (see Figure 2). Thus, the SHG intensity is a direct measure for the residual molecular film thickness on the substrate.

Figure 3 illustrates the results of the LD measurements performed with left circularly polarized (LCP; Figure 3 a) and right circularly polarized (RCP; Figure 3 b) light on racemic samples. At first, the initial SHG intensities of the as‐prepared BINOL films were measured for both LCP and RCP light in order to obtain initial values of the SHG intensities *I*_LCP_ and *I*_RCP_ respectively, and the normalized values are shown in the graphs. The pulse energy and focusing were adjusted to suppress the nonlinear optical response from the BK7 substrate (see Figure S2 in the Supporting Information), while keeping the optical intensities well below the photo‐damage threshold of BINOL.^8^

**Figure 3:**
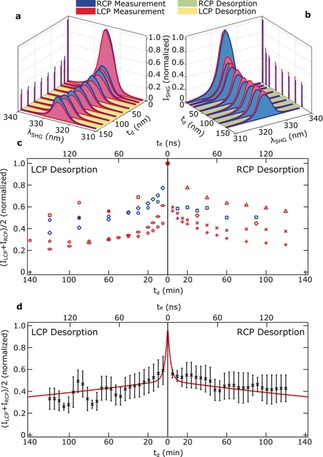
a,b) SHG‐spectra measured upon excitation with RCP (blue) and LCP (red) light at various desorption times (*t*_d_) are denoted as RCP/LCP measurement, respectively. Between SHG measurements, samples are exposed to LCP (a) or RCP (b) light for desorption. These steps are denoted as LCP/RCP desorption and are indicated with green and yellow shaded ground areas. The vertical lines indicate the normalized integrated intensities of the spectra (I_SHG_). c) Evolution of average SHG intensities recorded during desorption. Different symbols represent individual irradiation experiments carried out with 600 nm (blue) and 650 nm (red) on separate areas on several thin film samples. d) Moving average of the data in (c) weighted with Gaussian function. The red lines represent a biexponential fit (see text).

After measuring the initial SHG intensities, we desorbed BINOL from the sample by irradiation with circularly polarized light for a time *t*_d_. The respective light exposure time *t_e_* is the actual light–matter interaction time and takes into account the pulse duration of 50 fs and the repetition rate of the laser system of 1 kHz. The corresponding results of the LD of the racemic BINOL films during desorption by LCP and RCP are summarized in Figure 3 c. The SHG intensities depicted here are the average of the SHG signals generated by LCP and RCP light from the samples after each desorption step. Results for LCP desorption are shown on the left‐hand side and for RCP desorption on the right‐hand side of the graph. The large fluctuations of the data in Figure 3 c are caused by variations of the thickness and homogeneity of samples and fluctuations in laser intensity. Nonetheless, the measurement accuracy and spot‐to‐spot reproducibility of the desorption behaviour is symmetric with regard to the vertical axis of Figure 3 c. The average data from multiple experiments are shown in Figure 3 d. The results indicate that the overall desorption, that is, the sum of the desorption that occurs for (*R*)‐ and (*S*)‐BINOL, of the racemic film does not depend on the polarization handedness of light. The partial desorption of (*R*)‐ and (*S*)‐BINOL, however, is not necessarily identical.

The optical activity of the sample is determined from the measured SHG intensities and expressed by the anisotropy factor *g*=2(*I*_LCP_−*I*_RCP_)*/*(*I*_LCP_ + *I*_RCP_). It should be mentioned here that the initial values of *I*_LCP_ and *I*_RCP_ are not necessarily identical and thus the *g* value of the as‐prepared samples (*g*_0_) might deviate from zero. Although *g*_0_ varies from sample to sample, it stays unchanged without LD or when desorbed with linearly polarized light as is shown below. Since our aim in this communication is to look at the optical activity generated as the result of interaction with circularly polarized light, in order to make comparison between data from various measurements more reliable, we introduce Δ*g*=*g*−*g*_0_.

Before and after each desorption step we measured the optical activity of the film. We repeated the cycle of measurement‐desorption for at least 2 hours for all the samples. In fact, as is shown in Figure 4, the anisotropy factor Δ*g* of the remaining BINOL film fully correlates with the handedness of the light that has been used for LD. Figure 4 a demonstrates that despite the fluctuations in the laser intensity and variations in sample properties, the striking correlation between the handedness of the circularly polarized light used for LD and the emerging optical activity is consistent for every single measurement.

**Figure 4:**
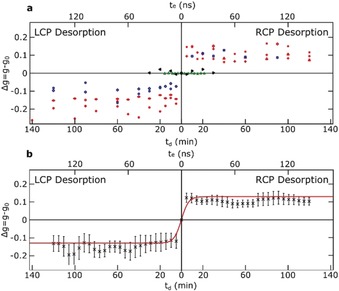
Changes in anisotropy factors at various desorption times. a) Different symbols represent the calculated Δg for data points in Figure 3 c. Red and blue symbols stand for excitation at 650 and 600 nm, respectively. Black symbols are the average Δ*g* for the desorption measurements done with linearly polarized light via the same setup settings as the data taken with circularly polarized light. Green symbols present the data for desorbing an achiral molecule (2‐naphthole) by LCP and RCP. b) Moving average of the data in (c). The red lines represent a biexponential fit (see text).

We observed symmetric intensity decrease (see Figure 3 c) and antisymmetric Δ*g* for LD with LCP and RCP light for all samples and at both wavelengths (600 and 650 nm) used in this study (see Figure 4 a). Negative test experiments on achiral 2‐naphthol samples show no Δ*g* generation, as expected (see Figure 4 a, green symbols). Our observations clearly show that desorption with circularly polarized light leads to preferential desorption of enantiomers of BINOL from an achiral surface, as witnessed by the resulting dependence of Δ*g* on handedness of light. This effect can be confidently assigned to the polarization handedness of the light since no Δ*g* is observed in the case of desorption with linearly polarized light (see black symbols in Figure 4 a). This also unambiguously demonstrates that the desorption channel of photoexcitation without subsequent thermal equilibration is opened up at the expense of thermal desorption.^9^ Accordingly, in the present case, LD cannot simply be considered to be equivalent to a thermal desorption at a high heating rate. The interaction of circularly polarized light with the enantiomers clearly plays a non‐negligible if not a dominant role in the process of LD.

Although in early days of LD electron‐ and photon‐stimulated processes, such as those suggested by Menzel, Gomer, and Redhead,^10^ and Antionewicz^11^ (quantum mechanical mechanisms), were considered to play a major role,^12^ currently the most commonly accepted mechanisms for LD are based on thermal desorption, where the photon energy is efficiently coupled with vibrational modes and phonons in molecular and solid state system, respectively. In this thermal picture, the polarization of the photons plays no role. The inclusion of quantum mechanical (QM) processes, however, is a core necessity for explanation of our observation, since only in this way the emergence of such a large anisotropy factor can be understood.

We have constructed a simplified phenomenological model, which could describe our observation (for details, see the Supporting Information). Briefly, in this model, we allow (*R*)‐ and (*S*)‐BINOL molecules to have different desorption rates caused by TPA when interacting with circularly polarized light. The desorption rates include thermal as well as QM contributions as mentioned above. The desorption process occurs at the surface layers. In contrast, the TPA of bulk BINOL molecules ends with a fast energy relaxation and subsequent local heating according to the model. Bulk heating occurs since excited molecules cannot evaporate from the film before collisional energy exchange if excited deep within the molecular film. These processes facilitate diffusion within the film, which might be derived from the compositional gradient between the bulk and the surface, if enantiospecific desorption occurs. This way, the depletion of the strongly desorbing enantiomer caused by LD is partially compensated from lower layers. As a result, the enantiomeric excess (*ee*) that is generated at the surface is transferred through the bulk of the film and the strongly desorbing enantiomer is continuously delivered to the surface for further LD.

As shown in Figure 3 d, the desorption curves indicate a fast initial desorption rate manifested by a rapid drop in the SHG intensity of the samples, which becomes slower for longer desorption times. Note that the SHG intensity is linearly proportional to the film thickness and thus to the number of BINOL molecules (see Figure 2). Consequently, the intensity change of the SHG signal directly monitors the change in the number of molecules, that is, total desorption rate. The correlation between the SHG intensity decay and material removal by desorption is supported by confocal microscopy images and profilometric data (see the Supporting Information). A biexponential fit in Figure 3 d shows a fast initial desorption rate (ca. 0.3 min^−1^), which becomes almost two orders of magnitude slower for longer desorption times (ca. 0.004 min^−1^). Although these rates depend on the used experimental conditions, their ratio (ca. 75) indicates an upper limit for the significant difference in the desorption rates of both enantiomers induced by circularly polarized light. We emphasize that cascaded second order nonlinearity, which is responsible for TPA process,^13^ possesses an even higher asymmetry than the SHG process.

Furthermore, the SHG intensity on Figure 3 d drops to 55 % of its initial value with the higher desorption rate. Looking at Figure 4 a, we see that in each case the anisotropy factor of the film almost saturates at the same time as the slow desorption rate sets in. Within this oversimplified model, this result suggests that the film has been almost purified enantiomerically very quickly by desorbing one enantiomer while largely leaving the other enantiomer in the film. Such a process would only be feasible if the molecules in the film are highly mobile, possessing a high diffusion coefficient. Apparently the TPA in the bulk accelerates the diffusion process so that the enantiomeric excess that is generated at the surface is quickly transferred through the bulk of the film. It should be noted that this oversimplified model, although self‐consistent, very likely overestimates the achieved *ee*.

In order to verify the absolute value of the achieved *ee* in the desorbed samples experimentally, knowledge of the anisotropy factors of the pure enantiomers *g_p_* determined by the same method are required. However, as was demonstrated very recently, unlike racemic films, enantiopure films of BINOL crystallize in optically active superstructures (see the Supporting Information) wherein the anisotropy factor of the film varies strongly and is very different from that of the constituent molecules. Consequently, measurements of *g_p_* with our setup are not reliable. We prepared samples with 10 % *ee* to calibrate the Δ*g* scale, with the assumption that at this enantiomeric ratio, the film properties would be close enough to the racemate rather than the pure enantiomer films. Although the 10 % *ee* samples do not resolve the calibration problem completely, they provide a more reliable value due to their much smaller distribution width. Taking the average of the values (*g*=0.069, for *ee*=10 %) as a point on the hypothetical calibration curve, we conclude that the generated *g* of 0.13 for the laser‐desorbed spots corresponds to an *ee* of 20 %. Earlier studies on nonlinear anisotropy factor of BINOL monolayers measured by SHG‐CD also report a value of 0.7 that might be used as *g*_p_.7b This value also leads to an averaged *ee* of only 20 %, which represents a lower limit of enantioenrichment.

The possibility of enantiospecific LD provides new and unprecedented opportunities. In this work, we report enantiospecific desorption by circularly polarized light. The achieved average *ee* lies between 20 % and >90 % according to the used estimations. We believe that by optimizing the process, for example, temperature, photon energy and time profile, and thickness of the film, and by introducing desorption–adsorption cycles, we may achieve a separation close to 100 % for other systems too. As a result of this work, an easy enantiomeric enrichment of racemic samples is now less remote. In the pharmaceutical industry, enantiospecific LD may become attractive because the risk of contamination through additional chemicals typically used for the separation of enantiomers is of no relevance. In heterogeneous asymmetric catalysis, enantiospecific LD may also become an important tool for the separation of nonvolatile reaction products.

## Conflict of interest

The authors declare no conflict of interest.

## Supporting information

As a service to our authors and readers, this journal provides supporting information supplied by the authors. Such materials are peer reviewed and may be re‐organized for online delivery, but are not copy‐edited or typeset. Technical support issues arising from supporting information (other than missing files) should be addressed to the authors.

Supplementary
