# Supplementary material for: Enantiospecific Desorption Triggered by Circularly Polarized Light
Source: Angew Chem Int Ed Engl. 2019 Sep 3;58(44):15685–9. doi: 10.1002/anie.201906630 (PMC6851867; doi:10.1002/anie.201906630)
Supplement: Supplementary file 1 — Supplementary [file ANIE-58-15685-s001.pdf]

Angewandte Chemie
Eine Zeitschrift der Gesellschaft Deutscher Chemiker

Supporting Information

Enantiospecific Desorption Triggered by Circularly Polarized Light

Farinaz Mortaheb[+], Katrin Oberhofer[+], Johann Riemensberger, Florian Ristow, Reinhard Kienberger, Ulrich Heiz, Hristo Iglev, and Aras Kartouzian**

anie_201906630_sm_miscellaneous_information.pdf

SUPPORTING INFORMATION

Desorption and SHG-CD experimental setup.

A regenerative Ti:sapphire amplifier system (CPA-2010, Clark-MXR) delivers 1 mJ, 150 fs pulses at a central wavelength of 775 nm and a repetition rate of 1 kHz. A fraction of 180 μJ is used to pump a two-stage non-collinear optical parametric amplifier (NOPA) (*2*). The irradiation pulses (600 and 650 nm, 0.6 -2.5 μJ) for the desorption experiment are generated from the linear polarized NOPA output pulses, which are compressed to durations below 50 fs close to Fourier-limit. Taking into account the diameter of the laser spot onto the sample of ≈ 250 μm, the peak intensity and flux in the desorption experiments are estimated to be 24 – 100 GW/cm^2 and 1.2 – 5.1 mJ/cm^2, respectively. A schematic diagram of the experimental setup is shown in Figure S1. A quarter-wave plate "QWP" alters the original linear polarization of the laser light to right circularly polarized (RCP) or left circularly polarized (LCP). Furthermore, the circularly polarized pulses are focused onto the sample by a lens "L1" (fused silica, f = 100 mm) causing desorption and second harmonic generation (SHG) simultaneously in the BINOL film and on the sample surface. The SHG is first co-propagating with the fundamental laser light through a collimating lens "L2" (fused silica, *f* = 40 mm), then separated from the latter in a pair of Brewster prisms "P". The SHG light is focused by a third lens "L3" (fused silica, *f* = 100 mm), passes through the bandpass filter "F" (Schott 2UG11, 1UG5) and impinges onto the detector (Horiba MicroHR, LN-CCD Princeton Instruments). We adjusted the experimental parameters in order to avoid any contribution to the SHG signal from the BK7 substrates, as shown in Figure S2.

Polarization resolved microscopy on S-, R-, and racemic BINOL films.

Figure S3 shows microscope images of R-, S-, and racemic BINOL films adsorbed at BK7 substrate. The images are recorded with a polarization resolved microscope (Leica DMI6000 B). The images reveal that unlike the racemic films (S3c), the enantiopure R-BINOL (S3a) and S-BINOL films (S3b) form characteristic crystal structures. Most important, these structures are optically active. Furthermore, the microscope image shows a pattern of brighter and darker areas when placed between two crossed polarizers (0°), i.e. microscopic regions exhibiting different optical activity. Rotating the analyzer around its normal axis with respect to the polarizer by -20° or +20° leads to a change of the pattern around the center of each crystal revealing a complex macroscopic chirality, as indicated for R-BINOL in Fig. S3a. Thus, the anisotropy factor measured by our SHG-CD setup shows a strong variation depending on the position and diameter of the exciting laser spot and its exact position on the surface. In addition, the anisotropy factor arises from the chiral crystalline structure, which is not necessarily the same as the microscopic g-value from a single molecule or small domain of the crystal.

Phenomenological model for asymmetric LD with circularly polarized light

In order to estimate the quantum mechanical (QM) nature of the desorption process, we developed a simple phenomenological model. In this model, we allow for R-BINOL and S-BINOL molecules to have different desorption rates caused by two photon absorption (TPA) when interacting with circularly polarized light. That is, with two different time independent desorption rates δ_1 and δ_2 for R-BINOL and S-BINOL molecules when desorbed with LCP, respectively. The excitation with the RCP leads to exchange of the corresponding desorption rates so that now S-BINOL take the desorption rate δ_1 and R-BINOL takes the desorption rate δ_2. These desorption rates include thermal as well as QM contributions. It should be emphasized that the values for δ_1 and δ_2 are not assumed

to be different but only allowed to be different. If the model leads to different values for δ_1 and δ_2 it can be concluded that QM desorption process plays an important role in LD since the thermal desorption will not distinguish between the enantiomers. The desorption process occurs at the surface layers. On the contrary, the TPA of bulk BINOL molecules ends in a fast energy relaxation and subsequent local heating according to the model. These processes facilitate the diffusion within the film which might be derived by the compositional gradient between the bulk and the surface, if enantiospecific desorption occurs. The double exponential behavior of the desorption curves in Figure 3d of the manuscript strongly supports the above scenario. As shown in the main manuscript (Figure 2) the SHG intensity is linearly proportional to the film thickness and thus to the number of molecules. This in turn means that the change of the intensity of the SHG signal directly monitors the change in the number of molecules, i.e. total desorption rate *d*. The total desorption rate is the sum of the individual desorption rates multiplied by the fraction of the enantiomers (C_R, C_S) at the surface as given in the following equation.

$$d = \delta_1 \cdot C_R + \delta_2 \cdot C_S = \boldsymbol{\delta_1 \cdot C_R + \delta_2 \cdot (1 - C_R)} = \frac{\Delta I_{SHG}}{\Delta t_d}$$

The initial conditions ($t_d=0,\ C_R = C_S=0.5$) model a racemic film. Hence the total desorption rate can change between $d_{(t_d=0)} = \frac{1}{2}(\delta_1 + \delta_2)$ for the racemic surface and $d = \delta_2$ for the fully purified surface ($C_R = 0$).

Fig. 3d shows a fast, initial desorption rate $d \cong 0.3\ min^{-1}$ (red line in Fig. 3d of the manuscipt), which is manifested by a rapid drop in the SHG intensity. The desorption rate becomes then almost two orders of magnitude slower for longer desorption times ($d \cong 0.004\, min^{-1}$). These rates depend on the used experimental conditions, however their ratio of about 80 indicates a significant difference in the desorption rates of both enantiomers induced by TPA of with circularly polarized

light. We emphasize, that cascaded second order nonlinearity which is responsible for TPA process, exhibit higher asymmetry than the included second-order processes as SHG. According to Fig. 3d, the SHG intensity drops to almost 55% of its initial value very quickly (with the quicker desorption rate). This means that almost the half of the racemic film is desorbed very quickly, while the other half of the film is desorbed with the slower rate. Looking at Fig. 4b and focusing on one side of the graph (left for desorption with LCP, and right for desorption with RCP light), we see that in each case the anisotropy factor of the film almost saturates at the same time as the slow desorption rate sets in. This suggests that the film has been purified enantiomerically very quickly by desorbing one enantiomer while largely leaving the other enantiomer in the film. Such a process would only be feasible if the molecules in the film are highly mobile possessing a high diffusion coefficient. Apparently the TPA induced bulk heating accelerates the diffusion process so that the depletion of the desorbing enantiomer can be compensated from lower layers fast enough. Bulk heating occurs as excited molecules cannot evaporate from the film before collisional energy exchange if excited deep within the molecular film.

Second harmonic generation spectrum of BINOL

The SHG spectrum of BINOL is shown in Fig. S4. It closely follows the linear absorption spectrum of the molecule in the probed range.

Time of flight of desorbed BINOL

Figure S5 shows a mass spectrum of desorbed BINOL measured by Time-Of-Flight. Desorption has been achieved with ns long laser pulses. This shows that BINOL can be desorbed as molecule by laser desorption without being destroyed by the photons.

Film thickness and laser spot depth

Figure S6 provides an analysis of the desorbed laser spot by a combination of confocal microscopy (Device: Leica Confocal Sp5, Objective: 40x oil lens NA = 1.25, Acquisition Program: Leica Application Suite – Advanced Fluorescence) (Fig. S6a) and optical profilometry (Device: Bruker DektakXT). The thickness of the film is about 1.3 µm (fig S6d) and the depth of the leaser-desorbed spot is about 0.7 µm (fig S6 b and c), which corresponds to about 50% material removal. The SHG signal of laser spots has also dropped by approximately 50 %. This is in agreement with the correlation between the SHG intensity of the film and the film thickness demonstrated in figure 2.

10% ee and enantiopure samples

We have performed measurements on enantiopure samples as well as samples with 10% *ee* of each enantiomer in an attempt to calibrate the correlation between the measured anisotropy factor and the achieved *ee* of the samples upon desorption. Figure S7 shows the distribution of more than 50 measurements in each case. It is believed that formation of domains in the enantiopure samples leads to unreliable g-value measurements. This also leads to extreme scattering of the data and to non-mirror image average values for the two pure enantiomers. The average g for R-BINOL is -0.491 while for S-BINOL it is +0.924. The same issue appears to be present for samples with 10% ee. Again, the average g value for samples with excess of R-BINOL is smaller than for samples with excess S-BINOL. It is often the case that the commercially available enantiomers come with different impurities, which could affect crystallization and crystal sizes and thus lead to the observed asymmetry. Since the width of the distribution is much smaller in case of 10% ee samples, these provide a better point on the calibration curve for relating the ee of the desorbed samples with their g-value.

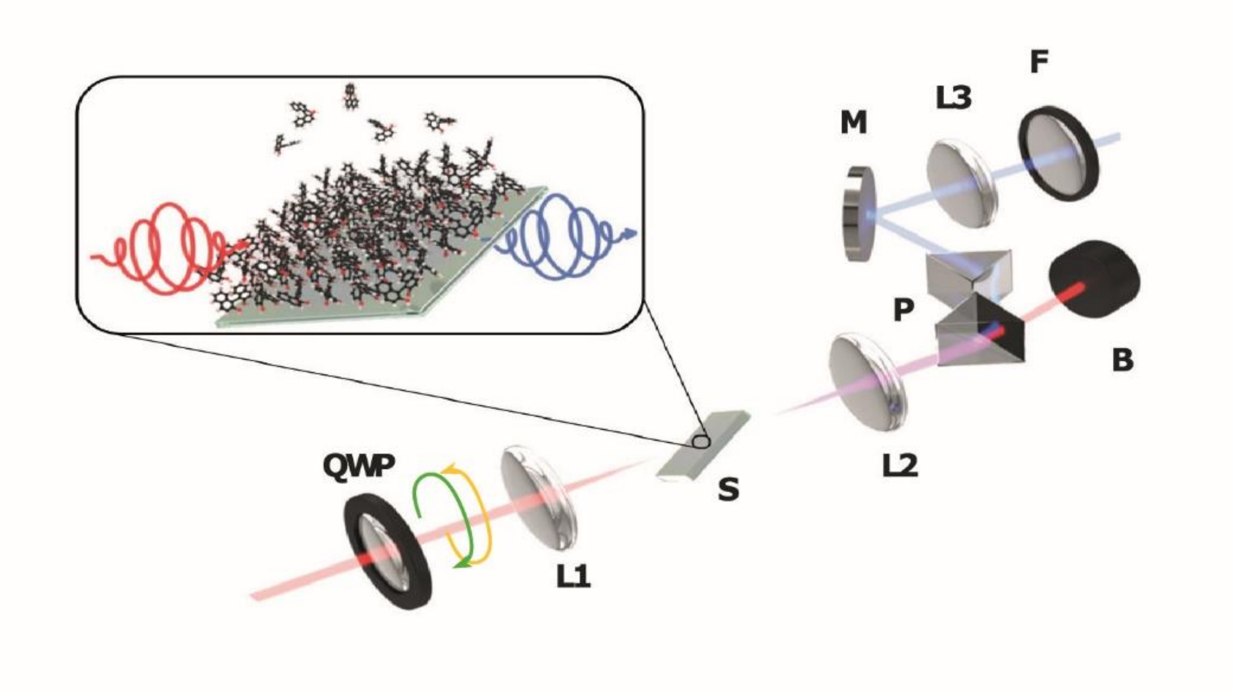

Figure S1**:** Experimental set-up. 50 fs laser pulses in the visible spectral range (red) are focused onto the sample shown in a magnified view on top, causing desorption of BINOL molecules. The quarter-wave plate (QWP) alters the original linear polarization of the laser light to RC (green) or LC (orange) polarization. SHG (blue light in the magnified) in the BINOL film and on the sample surface is collected at the detector.

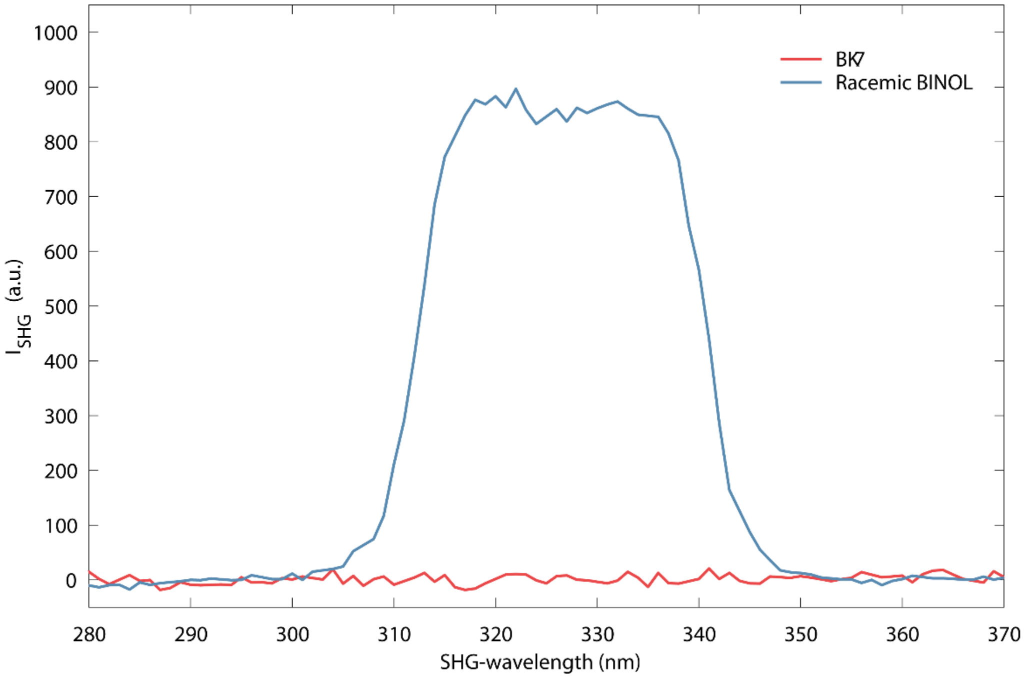

***Figure S2*:** SHG spectra from pure BK7 substrate and thin film of racemic BINOL. Evaporation performed onto similar substrates. SHG recorded with the identical experimental settings. No SHG signal from uncoated BK7 detectable.

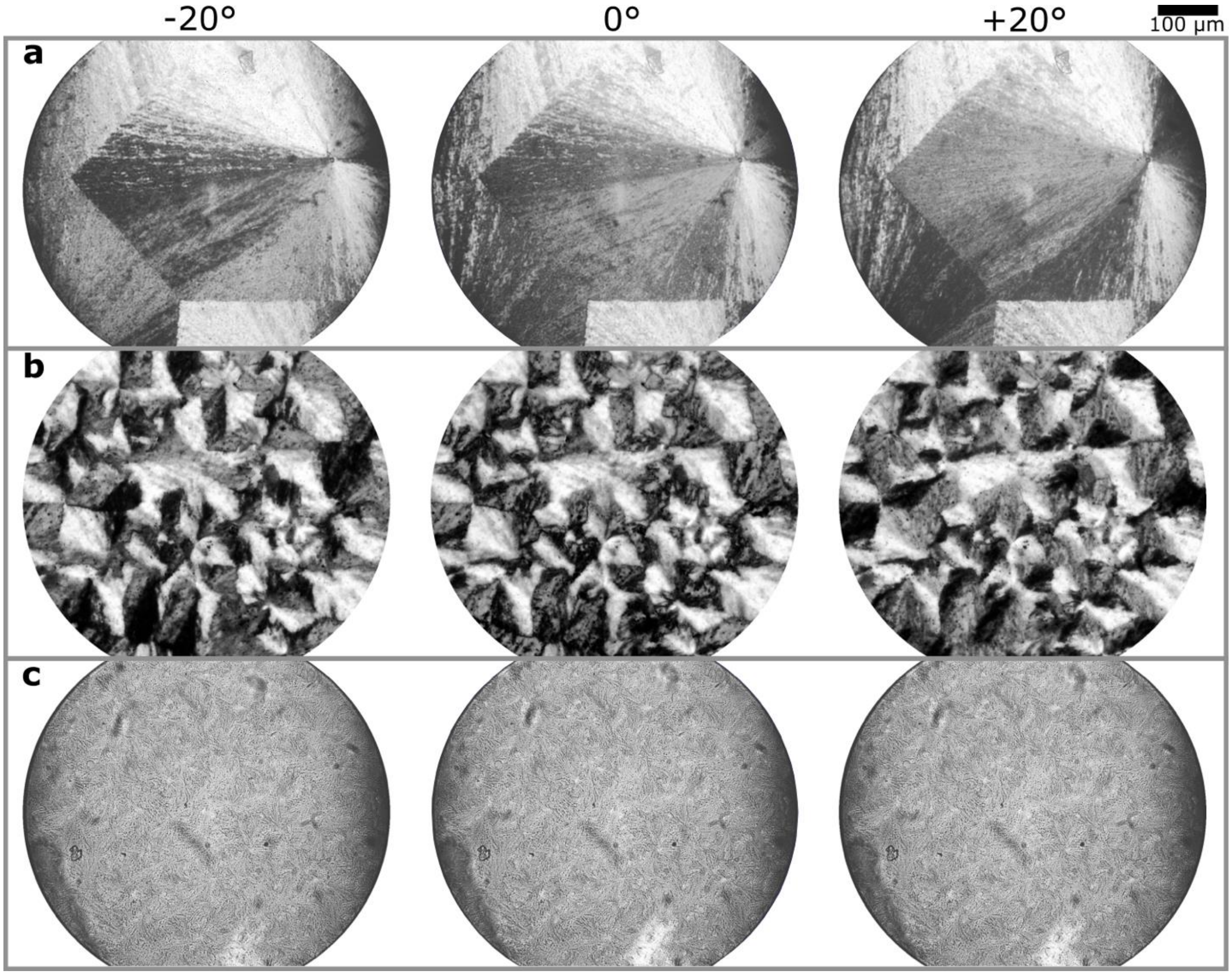

Figure S3**:** Polarization resolved microscopy on R-, S-, and racemic BINOL films on BK7 substrate. Images of R-BINOL (a), S-BINOL (b) and racemic film (c) measured with Leica DMI6000 B microscope (40x magnification). Analyzer is rotated by an angle of -20° (left), 0° (center) and +20° (right), with 0° being the position of minimum transmission.

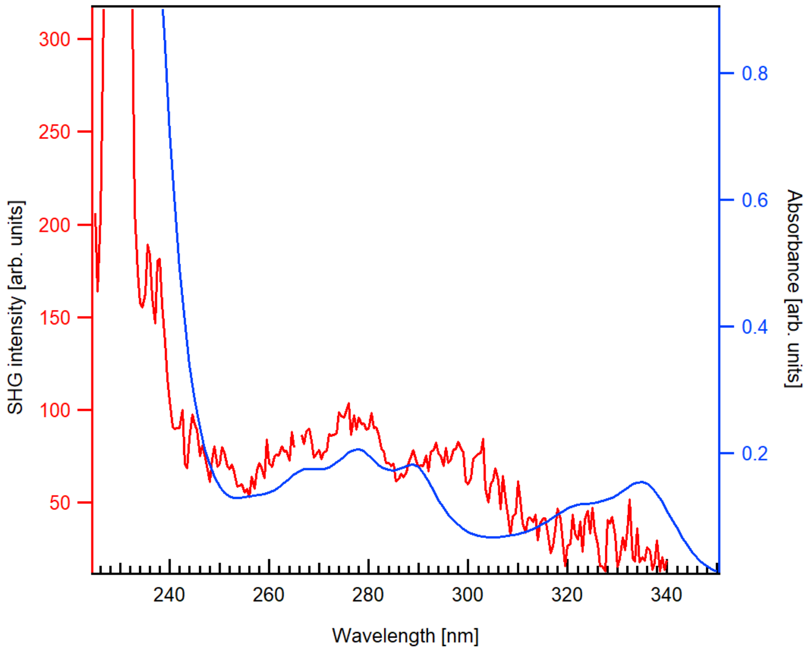

Figure S4: SHG spectrum of BINOL (red) and linear absorption spectrum of BINOL (blue).

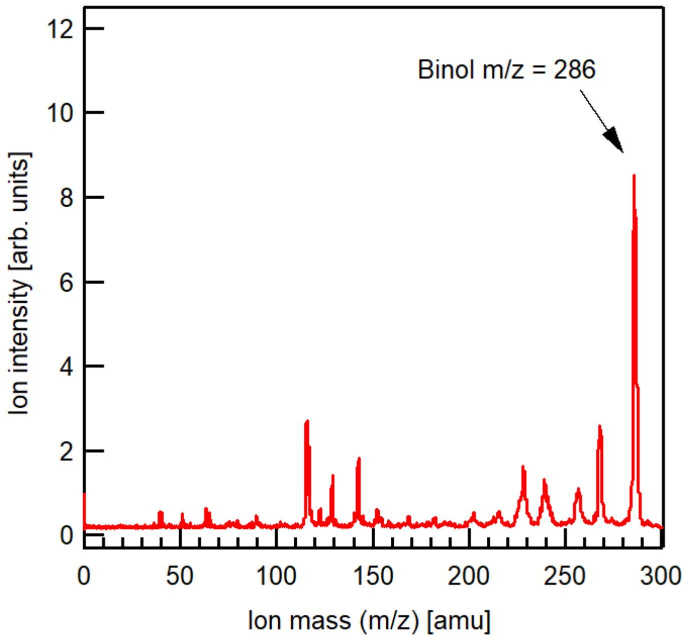

Figure S5: Mass spectrum of desorbed BINOL measured by time-of-flight with 1+1 ionization at 267.5 nm Desorption performed with 607 nm, 5 mJ, 10 ns pulses, focused down to a spot of 2 mm at , 30 Hz repetition rate.

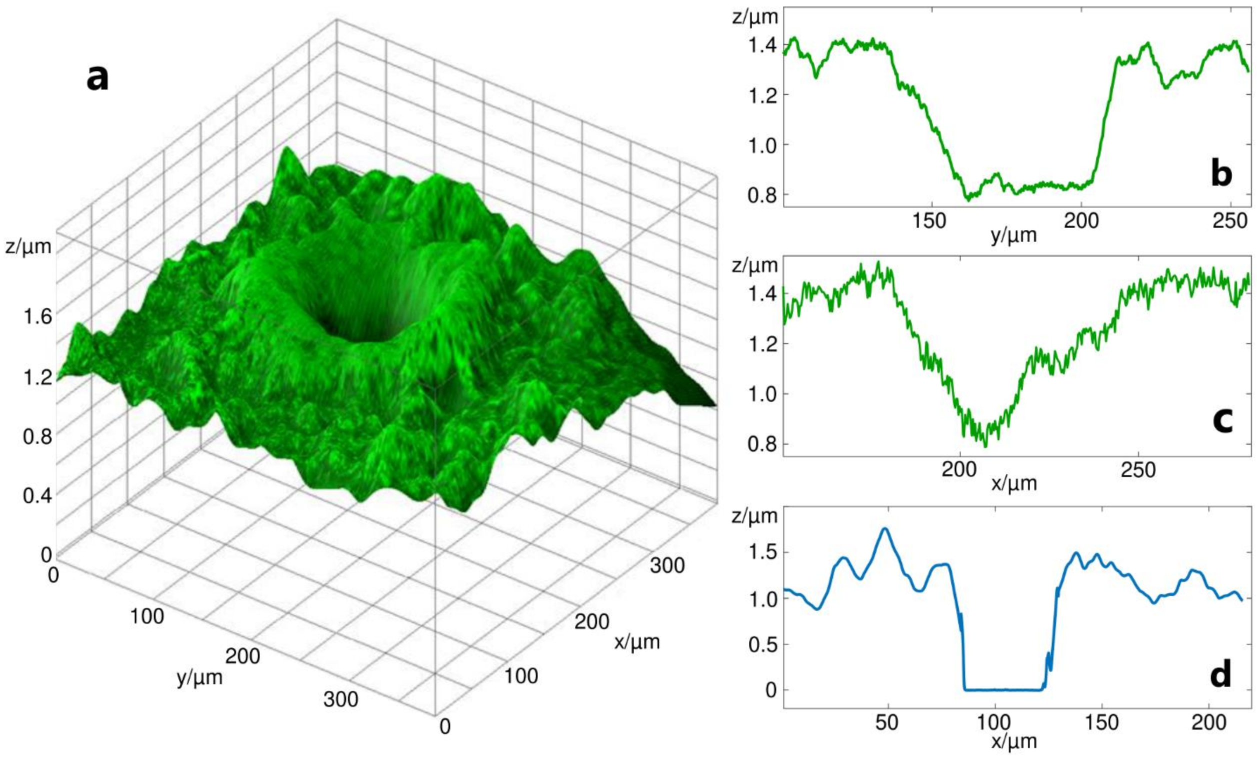

Figure S6**:** Confocal microscopy image of an LD spot (a) depth profile extracted from the microscopy image along x (b) and y (c) axis. (d) Thickness profile of a scratch on the film measured by profilometry, to verify the thickness of the film.

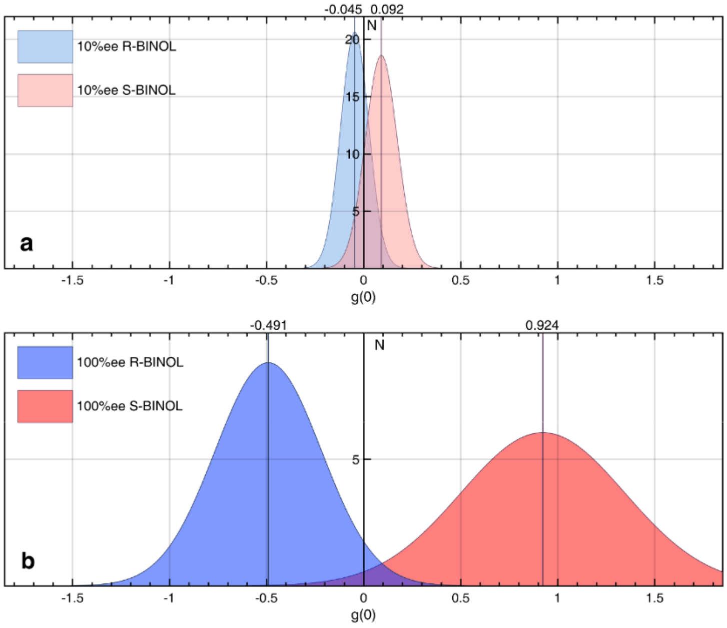

***Figure S7*: Distribution of the anisotropy factor, *g*, measured on 50 samples with 10% ee of each enantiomer (a) and on more that 100 enantiopure samples (b).
